# Altered Prostanoid Signaling Contributes to Increased Skin Tumorigenesis in Tpl2 Knockout Mice

**DOI:** 10.1371/journal.pone.0056212

**Published:** 2013-02-15

**Authors:** Kathleen L. DeCicco-Skinner, Sabrina J. Nolan, Monika M. Deshpande, Erika L. Trovato, Taylor A. Dempsey, Jonathan S. Wiest

**Affiliations:** 1 Department of Biology, American University, Washington, DC, United States of America; 2 Laboratory of Cancer Biology and Genetics, National Cancer Institute, National Institutes of Health, Bethesda, Maryland, United States of America; University of Connecticut Health Center, United States of America

## Abstract

Squamous cell carcinoma is the second most common form of skin cancer with the incidence expected to double over the next 20 years. Inflammation is believed to be a critical component in skin cancer progression. Therefore, understanding genes involved in the regulation of inflammatory pathways is vital to the design of targeted therapies. Numerous studies show cyclooxygenases (COXs) play an essential role in inflammation-associated cancers. *Tpl2 (MAP3K8)* is a protein kinase in the MAP Kinase signal transduction cascade. Previous research using a two-stage skin carcinogenesis model revealed that *Tpl2* ^−/−^ mice have significantly higher tumor incidence and inflammatory response than wild-type (WT) controls. The current study investigates whether cyclooxygenase-2 (COX-2) and COX-2- regulated prostaglandins and prostaglandin receptors drive the highly tumorigenic state of Tpl2^−/−^ mice by investigating the relationship between Tpl2 and COX-2. Keratinocytes from newborn WT or *Tpl2* ^−/−^ mice were treated with 12-*O*-tetradecanoylphorbol-13-acetate (TPA) for various times over 24 hours. Western analysis revealed significant differences in COX-2 and COX-2 dependent prostanoids and prostanoid receptors. Additionally, *in vivo* experiments confirmed that COX-2 and COX-2 downstream factors were elevated in TPA-treated Tpl2^−/−^ skin, as well as in papillomas from *Tpl2* ^−/−^ mice. Use of the selective COX-2 inhibitor Celecoxib showed the increased tumorigenesis in the Tpl2^−/−^ mice to primarily be mediated through COX-2. These experiments illustrate COX-2 induction in the absence of Tpl2 may be responsible for the increased tumorigenesis found in *Tpl2* ^−/−^ mice. Defining the relationship between Tpl2 and COX-2 may lead to new ways to downregulate COX-2 through the modulation of Tpl2.

## Introduction

Deregulation in mitogen activated protein kinase (MAPK) signaling is a common alteration in the development or progression of skin cancer [1]. *Tumor Progression Locus 2 (Tpl2),* also known as *MAP3K8*, is a MAP3K serine/threonine kinase in the MAPK signal transduction cascade [2]. *Tpl2* resides upstream of the MAPK ERK where it functions to phosphorylate MEK, the ERK kinase [3]. In addition to participating in ERK signaling, Tpl2 cross-talks with numerous other pathways including JNK, p38, NFAT, and nuclear factor kappa B (NF-κB) [4]–[8]. In non-stimulated cells, Tpl2 is held in complex with ABIN2 and the NF-κB precursor protein p105 [9]. This interaction stabilizes the Tpl2 protein but also prevents Tpl2 and NF-κB from activating their downstream signaling cascades by inhibiting the kinase activity of Tpl2 and the proteolysis of NF-κB p105 [10]. Upon activation of Tpl2 by various pro-inflammatory stimuli, IκB kinase (IKK) phosphorylates p105, releasing Tpl2 and p105 from the complex. This newly liberated Tpl2 now phosphorylates substrates in the ERK and JNK pathways. Additionally, p105 is subsequently degraded into p50 by the proteosome. p50 can now dimerize with other NF-κB family members and translocate to the nucleus where the active NF-κB complex can regulate over 400 genes. The overall result is an upregulation of diverse genes involved in growth, differentiation, and inflammation.

The *Tpl2/MAP3k8* gene was first isolated from thyroid tumors as a gene capable of inducing morphological transformation of NIH3T3 and SHOK cells [11]. Early reports in rodents showed *Tpl2* truncation of the C-terminus, resulting from provirus insertion, to be associated with T-cell lymphoma [12]. Subsequently several reports have found elevated *MAP3K8* activity in a number of human cancers including breast, endometrial, thymomas, lymphomas, lung, Hodgkin’s disease, and nasopharyngeal carcinoma [4], [12]–[15]. Additionally, recent reports correlate heightened *MAP3K8* expression levels with acquired resistance to drug therapy in melanoma [16]. However, the exact role of *Tpl2* in carcinogenesis has remained an enigma. Overexpression of *Tpl2/MAP3k8 in vivo* is weakly oncogenic and *Tpl2* mutations in humans are rarely found [12], [17]. However, recent evidence suggests that under certain conditions *Tpl2* may serve a tumor suppressor role. Tpl2^−/−^ mice, when crossed with the T cell receptor transgene, develop a high incidence of T cell lymphomas, whereas wild type mice remain cancer free [18]. Moreover, our laboratory recently reported that nearly 80% of Tpl2^−/−^ mice developed chemically induced skin tumors compared to 16% of wild type mice, providing the first evidence in a *de novo* cancer model that *Tpl2* may serve as a tumor suppressor [19]. Therefore, the role of Tpl2 in tumorigenesis is complex, as either overexpression or reduced expression of this gene can promote a tumorigenic state depending on the cancer type [17].

Numerous reports suggest inflammation in the microenvironment contributes to the development or progression of skin cancer [20]. Among other inflammatory enzymes, cyclooxygenases (COXs) play an essential role in inflammation-associated cancers [21], [22]. COXs catalyze the conversion of arachidonic acid (AA) to the intermediate product prostaglandin H_2_ (PGH_2_) [21], [22]. PGH_2_ can then be converted to the biologically active prostanoids PGE_2_, PGD_2,_ PGF_2α_, PGI_2_ and TXA_2_ through prostanoid synthases. Human cells contain two primary COX isoforms, namely COX-1 and COX-2 [21]. Both isoforms catalyze the same reaction, but differ in their expression patterns. *COX-1* is expressed constitutively in most tissues, and is involved in a number of normal physiological processes, including maintenance of the gastric mucosa, platelet aggregation, and regulation of renal blood flow [22]. In contrast, *COX-2* expression is undetectable in most normal tissues but highly inducible. It is expressed rapidly and transiently in response to inflammatory or mitogenic stimuli. Elevation in *COX-2 *mRNA and protein levels has been documented in cancers of the prostate, colon, breast, lung, cervix, pancreas, skin, intestine, and stomach [23]–[26]. Additionally, constitutive overexpression of COX-2 has been observed in chemically-induced papillomas and carcinomas [27].

In keratinocytes COX-2 exerts much of its effects through its product PGE_2_ [28]. PGE_2_ is the major prostaglandin produced in skin where it has numerous functions including wound healing, keratinocyte proliferation and localized edema [29]–[30]. It acts on skin cells in an autocrine or paracrine manner due to its rapid metabolic breakdown. Additionally, PGE_2_ has been reported to autoregulate its own synthesis by transcriptionally activating *COX-2* in a cAMP-dependent manner [30]. Although PGE_2_ facilitates skin homeostasis, it can also act as a tumor promoter, causing many of the hallmarks characteristic of cancer cells. Several reports have shown that overexpression of PGE_2_ increases tumor cell growth and progression [29]–[31]. Increased binding of cAMP response element binding protein (CREB), activator protein-1 (AP-1) and NF-κB to the promoter regions of cyclin D1 and vascular endothelial growth factor *(*VEGF*)* may be partially responsible for this heightened tumorigenesis [29]–[31].

PGE_2_ manifests its biological activity by binding to four different transmembrane receptors; EP1, EP2, EP3 and EP4 [23]. These G-protein coupled receptors appear to have different binding affinities for PGE_2,_ different downstream signaling pathways, and are differentially expressed in tissues and cells [23]. EP1 receptors are coupled to G_q_ and when activated increase intracellular calcium levels. Conversely, EP3 receptors are most often linked to G_i_ proteins and therefore can inhibit cAMP production. EP2 and EP4 receptors, by binding G_s_ proteins, are both coupled to adenylate cyclase (AC) [29], [32]. Activation of AC results in the conversion of ATP to cyclic AMP (cAMP) which in turn binds to protein kinase A (PKA), NF-κB, or CREB. Deregulation of cAMP pathways and aberrant activation of cAMP-controlled genes is linked to carcinoma growth, angiogenesis and resistance to apoptosis [33].

The purpose of this study was to determine whether COX-2 and COX-2- regulated prostaglandins and prostaglandin receptors drive the highly tumorigenic state of Tpl2^−/−^ mice by investigating the relationship between Tpl2 and COX-2. In this article we show that Tpl2^−/−^ mice have increased expression of cyclooxygenase-2 (COX-2) and COX-2- downstream factors including PGE_2,_ EP2, EP4, and cAMP. Additionally, through the use of the selective COX-2 inhibitor Celecoxib, we found the increased tumorigenesis in the Tpl2^−/−^ mice to be mediated through COX-2. These experiments illustrate that COX-2 induction in the absence of Tpl2 may be responsible for the increased tumorigenesis found in Tpl2^−/−^ mice.

## Materials and Methods

### Wildtype and Transgenic Mice

Male and female Tpl2^−/−^ mice were engineered as previously described [12]. C57BL/6 wildtype control mice were generated from the same colony as the Tpl2^−/−^ mice. All mice were bred and maintained at the NIH Animal Facility (Bethesda, MD). Tpl2^−/−^ status was regularly confirmed by PCR. All animal work was performed following NIH guidelines under an approved animal protocol.

### Immunoblotting

Primary keratinocytes and fibroblasts were isolated from Tpl2^−/−^ and C57Bl6 mice pups at 1–2 days of age as previously described and plated in 6 well dishes [34]. At the time of treatment, cells received 10 ng/ml 12-*O*-tetradecanoylphorbol-13-acetate (TPA) or Dimethyl Sulfoxide (DMSO) as the vehicle control. For the EP receptors, lysates were collected after 1, 3, 6, 12 and 24 hours of TPA treatment. For the COX-2 immunoblot, keratinocytes were primed with 5, 10, or 20 µM of the NF-κB inhibitor SN50 (Calbiochem, San Diego, CA) for 30 minutes prior to TPA treatment and again at time of TPA treatment. Total protein lysates were collected 18 hours post TPA treatment. For the H-ras blot, lysates were collected from WT or Tpl2^−/−^ keratinocytes prior to and five days after infection with the v-ras^Ha^ retrovirus. Lysates were prepared using M-PER reagent (Thermo Fisher Scientific, Rockford, IL) containing complete protease inhibitor (Roche, Indianapolis, IN) and Halt phophastase inhibitor (Thermo Fisher Scientific, Rockford, IL) in accordance with the manufacturer’s protocol. Proteins were separated using NuPAGE 4–12% Bis-Tris gradient gels (Invitrogen, Carlsbad, CA), then electrophoretically transferred onto PVDF membrane followed by immunoblotting. Membranes were incubated overnight at 4°C with EP1, EP2, EP3, β-actin (1∶1000) or EP4 (1∶400) primary rabbit antibodies (Cayman Chemical Company, Ann Arbor, MI), or H-ras antibody (1∶1000; EMD Millipore, Billerica, MA). Anti-rabbit HRP secondary antibody was applied at a dilution of 1∶2000 (Cell Signaling, Danvers, MA). West Dura Cemilluminescent substrate (Thermo, Rockland, Il) was used for signal detection. Membranes were visualized using a ChemiDoc-It imaging system (UVP, Upland, CA). Densitometry was performed on all western blots using the Image J program and signal normalized to β -actin.

### Immunohistochemistry

Papillomas and mouse skin treated with TPA for 4, 8, 12, or 24 hours were fixed in 10% normal buffered formalin (NBF) overnight and processed into paraffin blocks from which 4 µm sections were cut and stained with hematoxylin and eosin (H&E). The slides were rehydrated followed by antigen retrieval and endogenous peroxidase activity was quenched using methanol and hydrogen peroxide. Following washing and protein block (DAKO, Carpinteria, CA) the slides were incubated overnight at 4°C with COX2, EP2 or EP4 primary antibodies at dilutions ranging from 1∶200 to 1∶500 (Cayman Chemical Company, Ann Arbor, MI). Negative controls were acquired by substituting primary antibody with buffer. Following washing, the slides were incubated for 40 minutes at room temperature with Donkey anti-Goat biotinylated secondary antibody (Thermo Scientific, Rockford, IL). ABC reagent (Vector Labs Inc, Burlingame, CA) was then applied for amplification of primary antibody binding and 3,3-diaminobenzidine (DAB) was applied for visualization. Sections were melanin-bleached, and counterstained with Gill’s hematoxylin. The sections were dehydrated through graded alcohols, immersed in xylene, and mounted with coverslips. For the immunohistochemistry studies for EP2 or EP4, mice were treated with TPA for 0, 4, 8 12, 24, or 48 hours (n = 4/group). A minimum of four sections were stained for each time point and staining was repeated a minimum of three times. Representative areas were photographed using an Eclipse E800 digital camera (Nikon, Melville, NY) at 10× magnifications.

### PGE_2_ Assay

Wildtype or Tpl2^−/−^ keratinocytes received vehicle (DMSO) or TPA (10 ng/ml). Supernatants were collected after 30 or 60 minutes of TPA treatment. PGE_2_ concentration was determined using a PGE_2_ immunoassay kit from R&D Systems (Minneapolis, MN) per manufacturer’s instructions. Briefly, supernatants from control or treated keratinocytes were plated in a 96-well plate. Other wells received buffer only or standards supplied by the manufacturer. PGE_2_ antibody solution was added to wells followed by PGE_2_ conjugate and the plate was incubated for 2 hours. After four washes, substrate was added to wells and the plate was incubated for 30 minutes. Stop solution was added to each well and absorbance was read at 570 nm using a MultiSkan FC microplate reader (Thermo Scientific, Rockford, IL). The concentration of PGE_2_ was calculated from a standard curve and normalized to total cell number. The results presented are averages from triplicate samples ± standard deviation of two independent experiments.

### cAMP Assay

Five million wildtype or Tpl2^−/−^ keratinocytes received vehicle (DMSO) or TPA (10 ng/ml). Protein lysates were isolated after 60 minutes of TPA treatment. A cAMP assay was performed per manufacturer’s instructions using a kit from R&D Systems (Minneapolis, MN). Briefly, lysates from control or treated keratinocytes were plated in a 96-well plate. Other wells received buffer only or standards provided by the manufacturer. cAMP conjugate was added to wells followed by cAMP antibody solution and the plate was incubated for 2 hours. After three washes, pNPP substrate was added to wells and the plate was incubated for one hour. Stop solution was added to each well and absorbance was read at 570 nm using a MultiSkan FC microplate reader (Thermo Scientific, Rockford, IL). The concentration of cAMP was calculated from a standard curve. The results presented are averages from triplicate samples ± standard deviation of two independent experiments.

### Grafting Cells on Athymic Nude Mice

Tpl2^−/−^ or wildtype (WT) primary keratinocytes isolated from newborn mice were infected with the v-ras^Ha^ retrovirus, then trypsinized and used for grafting on day 8 as described previously [34]. Briefly, 24 eight week old athymic nude mice were equally divided into four groups (n = 6) and a graft site was prepared on their backs. Mice received six million keratinocytes (either WT or Tpl2^−/−^) mixed with six million fibroblasts (either WT or Tpl2^−/−^). The four groups of mice received the following combinations: a) wildtype keratinocytes plus wildtype fibroblasts, AIN-93G diet, b) wildtype keratinocytes plus wildtype fibroblasts, AIN-93G diet +500 ppm Celecoxib, c) Tpl2^−/−^ keratinocytes plus Tpl2^−/−^ fibroblasts, AIN-93G diet, or d) Tpl2^−/−^ keratinocytes plus Tpl2^−/−^ fibroblasts, AIN-93G diet +500 ppm Celecoxib. Tumor measurements began 10 days after grafting and continued once a week for 28 days. Tumor volume was calculated using the height multiplied by the length along the spinal column and the width at right angles to the length. Data are expressed as means ± SEM of approximate tumor volume in mm^3^.

### Statistical Analysis

PGE_2_, grafting data and cAMP levels were analyzed through two-way ANOVA analysis with PASW Statistics software (IBM, Armonk, NY). P-values <0.05 were considered significant and LSD Post-hoc tests were performed to identify the significant interaction.

## Results

### TPA-treated Mouse Skin and TPA-treated Keratinocytes from Tpl2^−/−^ Mice have Elevated COX-2 Signaling

**Figure 1:**
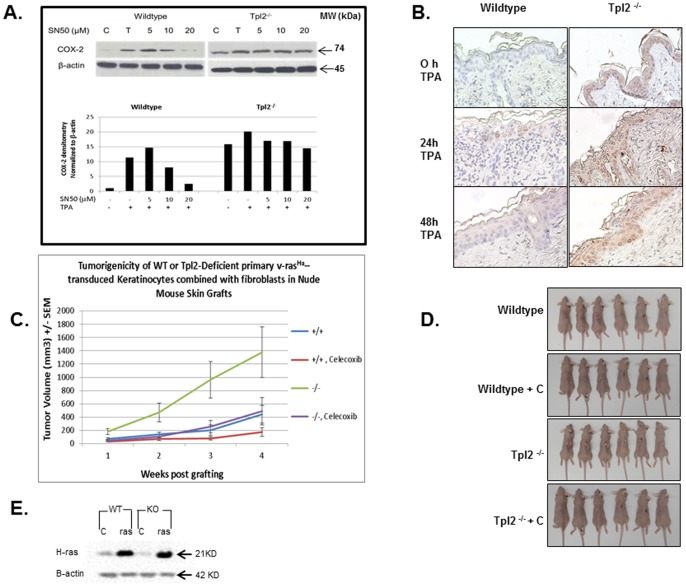
COX-2 signaling is augmented in keratinocytes and skin from Tpl2^−/−^ mice. (A) Primary keratinocytes were treated with DMSO control (Lane C), TPA (Lane T) (10 ng/ml), or TPA +5 µM, 10 µM, or 20 µM of the NF-κB inhibitor, SN50, and COX-2 expression measured 18 h later by western blot analysis. Densitometry of COX-2 after normalizion to β -actin is shown. (B) Immunohistochemical analysis of COX-2 in wildtype (WT) and Tpl2^−/−^ mouse skin. Normal WT and Tpl2^−/−^ skin as well as TPA-treated skin (24 or 48 hours) from WT and Tpl2^−/−^ mice was stained for COX-2 (1∶200 dilution). Magnification  = 10X. (C) Tumorigenicity of keratinocytes expressing oncogenic v-ras^Ha^ in nude mouse grafts. Data are expressed as means +/− SEM of approximate tumor volume in mm^3^. Statistical differences were found between mice grafted with v-ras^Ha^ transformed WT (+/+) or those grafted with v-ras^Ha^ transformed KO (−/−) keratinocytes as well as between mice grafted with Tpl2^−/−^ keratinocytes and those grafted with Tpl2^−/−^ keratinocytes and fed 500 ppm Celecoxib in their diets (p<0.01). No statistical difference was found when comparing mice grafted with Tpl2^−/−^ keratinocytes and fed Celecoxib and mice grafted with WT cells. (D) Clinical appearance of mice grafted with v-ras^Ha^ transformed WT or v-ras^Ha^ transformed Tpl2^−/−^ keratinocytes fed AIN-93G diet +/−500 ppm Celecoxib. (E) Western blotting of basal H-ras levels of WT or KO keratinocytes and H-ras levels post ras^Ha^ retrovirus transduction.

We recently reported Tpl2^−/−^ mice have increased inflammatory responses and this is primarily mediated through exaggerated NF-κB signaling [19]. COX-2 is an NF-κB regulated gene whose elevated expression is documented in a variety of neoplastic tissues. Using western analysis we assessed the basal expression of COX-2 in keratinocytes from wildtype and Tpl2^−/−^ mice. Basal expression of COX-2 is nearly 15 fold higher in Tpl2^−/−^ keratinocytes compared to wildtype cells, whose expression is barely detectable (Figure 1A). As expected, TPA treatment induced COX-2 protein in both genotypes. However, in wildtype mice this induction could be reversed in a dose-dependent manner when SN50, an NF-κB inhibitor, was added to the keratinocytes. In knockout mice, the heightened COX-2 expression remained high upon the addition of SN50, only beginning to slightly decrease with the highest dose of SN50. Additionally, using immunohistochemistry we assessed the level of COX-2 in TPA-treated mouse skin and found significant increases in Tpl2^−/−^ skin treated with TPA for 24 or 48 hours compared with skin from control animals (Figure 1B).

### Celecoxib Inhibits Tumors Developed on Nude Mice with v-ras^Ha^ Transduced Tpl2^−/−^ Cells

To show an upregulation in COX-2 correlates with the heightened tumorigenesis found in Tpl2^−/−^ mice, we grafted nude mice with v-ras^Ha^ –transduced keratinocytes from Tpl2^−/−^ or wildtype mice mixed with fibroblasts from the same genotype, and fed a subset of these animals the COX-2 selective inhibitor Celecoxib. We found nude mice grafted with v-ras^Ha^ transduced keratinocytes and fibroblasts from Tpl2^−/−^ mice developed four times larger tumors than mice grafted with v-ras^Ha^ transduced keratinocytes and fibroblasts from wildtype animals (Figure 1C, 1D). Administration of Celecoxib to nude mice grafted with v-ras^Ha^ transduced keratinocytes and fibroblasts from Tpl2^−/−^ mice decreased the size of the tumors to a level that was indistinguishable from untreated control mice. These differences were statistically significant (p<0.01). However, there were not statistical differences in tumor sizes between mice fed Celecoxib and grafted with Tpl2^−/−^ keratinocytes and mice grafted with wildtype keratinocytes (Figure 1C, 1D). The level of v-ras^Ha^ expression in each genotype following transduction was confirmed by Western blot (Figure 1E).

### Tpl2^−/−^ Keratinocytes have Enhanced PGE_2_ Signaling

**Figure 2:**
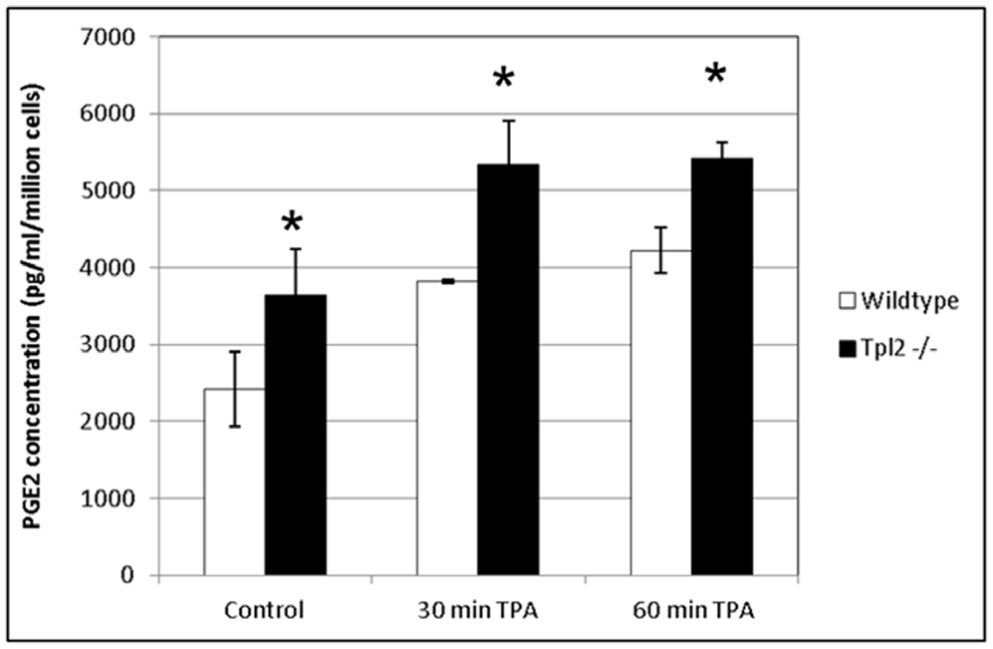
PGE_2_ production is higher in keratinocytes from Tpl2^−/−^ mice. Wildtype or Tpl2^−/−^ keratinocytes received vehicle (DMSO) or TPA (10 ng/ml). Supernatants were collected after 30 or 60 minutes of TPA treatment. PGE_2_ concentration was determined using a PGE_2_ immunoassay kit. Significant differences were found between genotypes at all time points (p<0.003).

COX-2 catalyzes the first committed step in prostanoid synthesis, resulting in the production of several prostaglandins including PGE_2_, the primary prostanoid found in skin. To assess whether Tpl2^−/−^ keratinocytes had elevated PGE_2_ levels, an immunoassay was performed. We found basal production of PGE_2_ was 35% higher in Tpl2^−/−^ keratinocytes than in wildtype cells (Figure 2). TPA treatment for 30 or 60 minutes exaggerated PGE_2_ release in both genotypes, but the PGE_2_ released from Tpl2^−/−^ keratinocytes remained significantly (p<0.003) greater than in wildtype cells.

### Tpl2^−/−^ Keratinocytes have Increased Expression of the PGE_2_ Receptors EP2 and EP4

**Figure 3:**
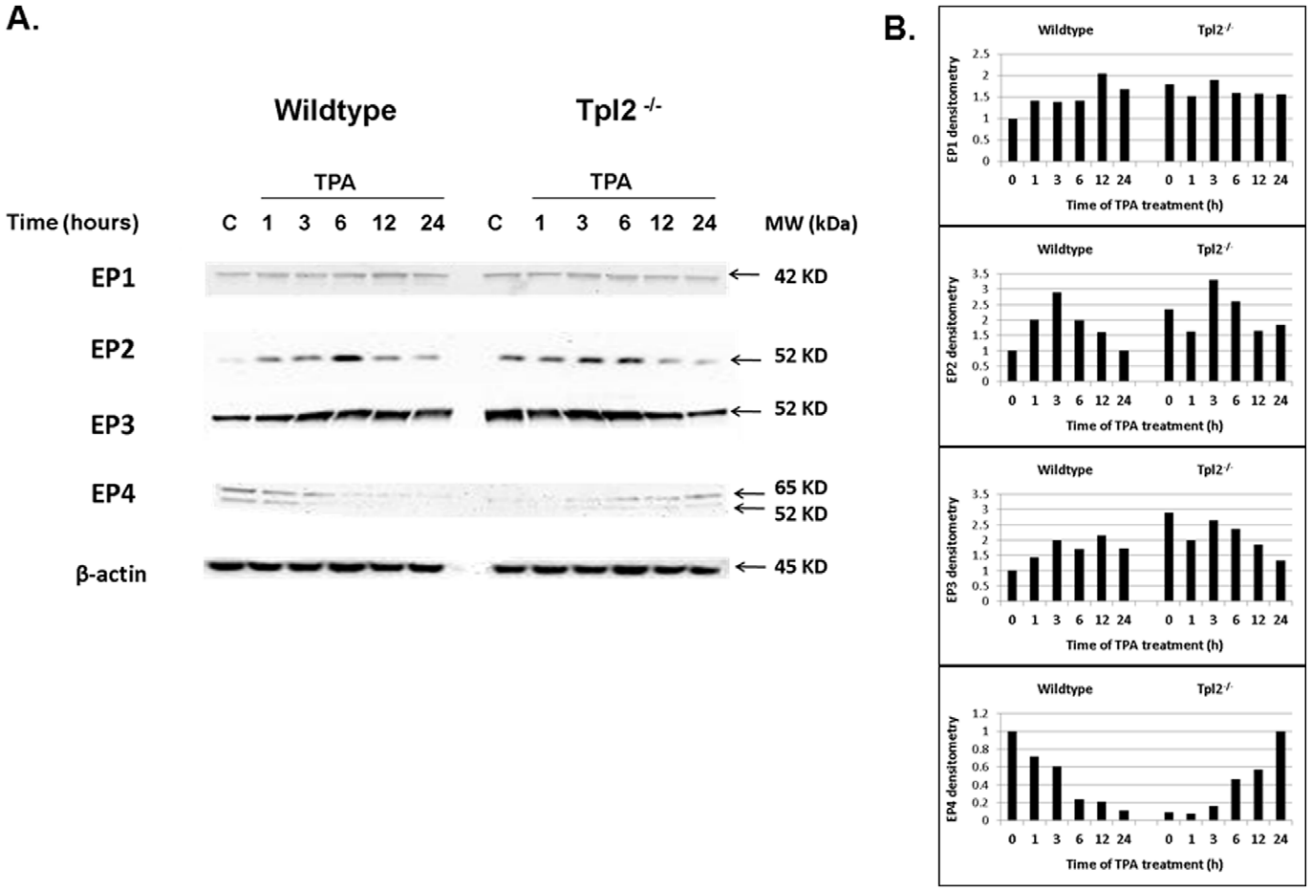
Western blotting of prostanoid receptor expression in wildtype and Tpl2^−/−^ mouse keratinocytes. Wildtype or Tpl2^−/−^ keratinocytes received DMSO only (labeled C) or TPA (10 ng/ml) for 1, 3, 6, 12, or 24 hours. Western blotting analysis was used to determine the levels of EP1, EP2, EP3, EP4, and β -actin. Bands were quantified through densitometry (Image J) and normalized to β -actin.

PGE_2_ is a major product of COX-2 activation and binds to one of four G-protein coupled receptors. Activation of two of these receptors, EP2 and EP4, is linked to increased skin tumor development and cancer cell migration [29], [35]. Western analysis with TPA-treated keratinocytes assessed if EP2 and EP4 receptors are differentially regulated in our wildtype and Tpl2^−/−^ model (Figure 3). We found Tpl2^−/−^ keratinocytes had 2.5 times higher basal expression of EP2 when compared to keratinocytes from wildtype mice. Interestingly, we also found TPA-treatment increased expression of EP4 in Tpl2^−/−^ keratinocytes, whereas TPA-treatment decreased expression of EP4 in wildtype cells. It is interesting to note that EP3 has been reported to oppose EP2 and EP4 as induction of EP3 receptor decreases cAMP [29], [32]. Our data suggests EP3 may work in an opposing fashion to EP4 in our model. As the levels of EP4 increase in our Tpl2^−/−^ cells the levels of EP3 decline. We found no differences in the expression of EP1, agreeing with previous literature that suggests EP1 does not have a major role in skin tumor formation [35].

### TPA-treated Skin Sections from Tpl2^−/−^ Mice Exhibit Increased EP2 and EP4 Expression

**Figure 4:**
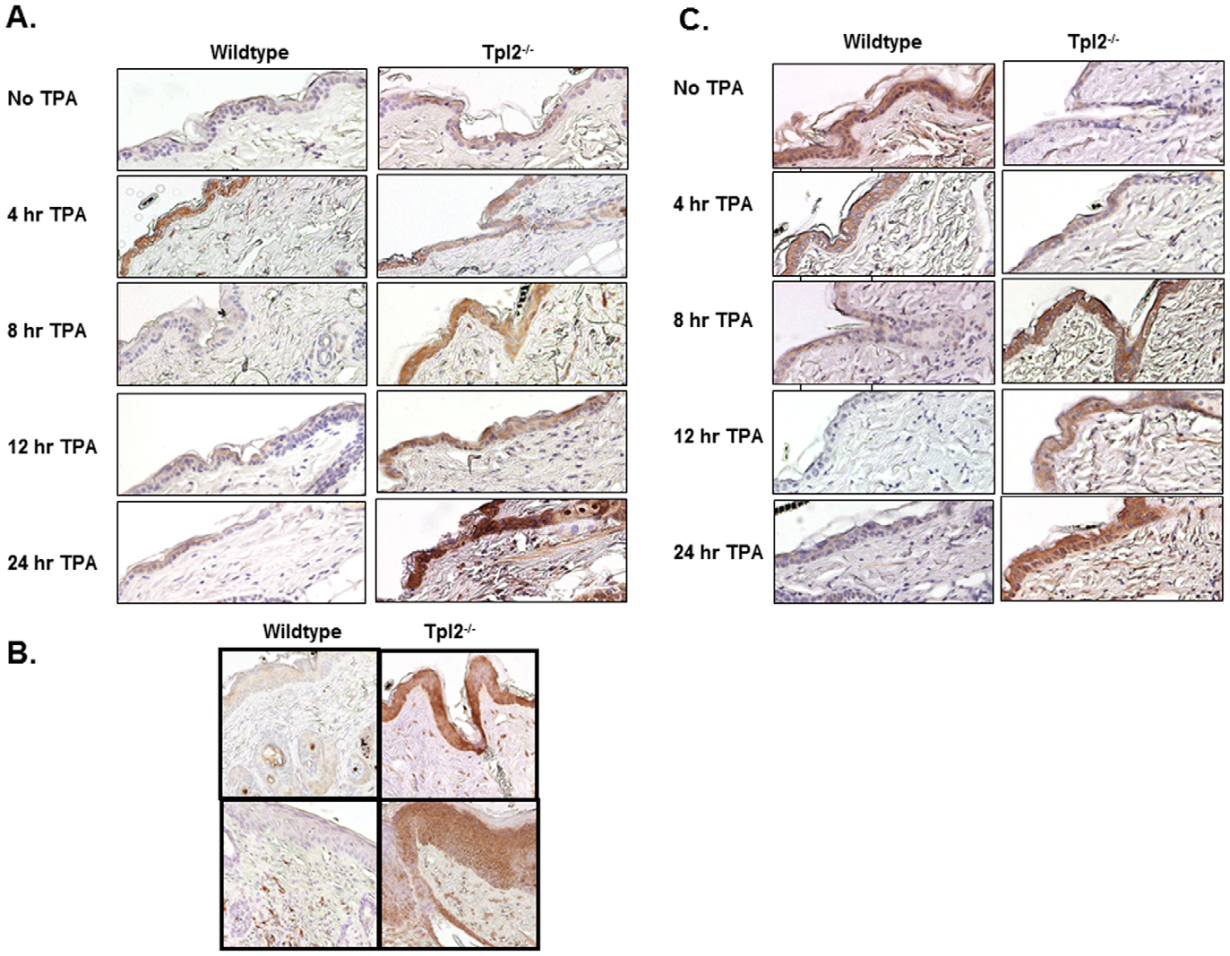
EP2 and EP4 receptor expression is heightened in epidermis and papillomas from Tpl2^−/−^ mice. Shaved wildtype and Tpl2^−/−^ mice were treated with TPA (10 µg) for 0, 4, 8, 12, or 24 hours and analyzed for expression of EP2 (Figure 4A) or EP4 (Figure 4C). Additionally, papillomas from wildtype and Tpl2^−/−^ mice were analyzed for expression of EP2 (Figure 4B). Magnification = 10X.

We performed immunohistochemistry to correlate our *in vitro* results with the expression levels of EP2 and EP4 in TPA-treated mouse skin and papillomas. Within 8 hours of TPA treatment, there was markedly higher staining of EP2 in Tpl2^−/−^ skin and this heightened staining was maintained over the course of 24 hours (Figure 4A). However, the wildtype mice showed relatively weak basal staining with an induction at the 4 hour time point. By 8 hours this induction has subsided. Additionally, we found higher levels of EP2 in papillomas from Tpl2^−/−^ mice supporting its role in tumorigenesis (Figure 4B). EP4 expression in TPA-treated wildtype and Tpl2^−/−^ mouse skin was also similar to what was observed *in vitro* in mouse keratinocytes. In wildtype mice, expression of this receptor is highest in untreated skin and declines with TPA treatment, being nearly undetectable after 8 hours of TPA treatment (Figure 4C). Expression of EP4 is low at basal levels in Tpl2^−/−^ mice but increases with TPA treatment over the course of 24 hours.

### TPA-treated Keratinocytes from Tpl2^−/−^ Mice Show Elevated cAMP Levels

**Figure 5:**
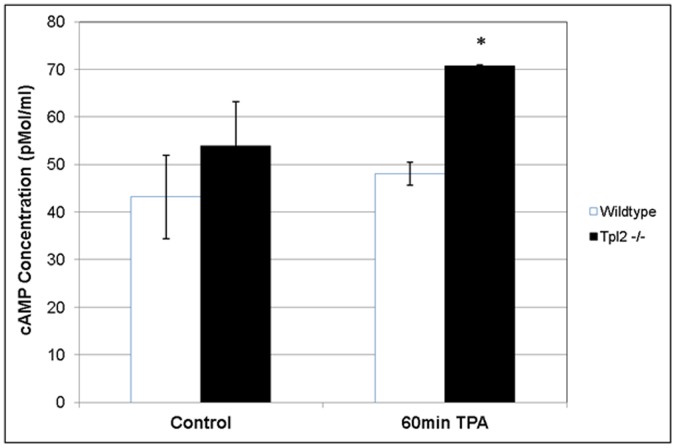
cAMP production is higher in keratinocytes from Tpl2^−/−^ mice. Wildtype or Tpl2^−/−^ keratinocytes received DMSO only (control) or TPA (10 ng/ml). Lysates were collected after 60 minutes of TPA treatment. cAMP concentration was determined using a cAMP immunoassay kit. Significant differences were found between genotypes following TPA treatment (p<0.002).

As demonstrated both *in vitro* and *in vivo*, Tpl2^−/−^ mice display heightened EP2 and EP4 receptor levels upon stimulation with TPA. As previously reported, EP2 and EP4 activation increases cAMP levels [29], [32]. As a second messenger, cAMP can induce numerous genes involved in cancer growth and progression [33]. We performed an immunoassay to determine if keratinocytes from our Tpl2^−/−^mice had elevated cAMP levels compared to keratinocytes from wildtype mice. Although basal cAMP levels were approximately 20% higher in untreated Tpl2^−/−^ keratinocytes, the differences were not significant. However, the level of cAMP produced by TPA-treated Tpl2^−/−^ keratinocytes was 46% higher than the level of cAMP produced by TPA-treated WT keratinocytes (70.8 pmol/ml vs. 48.4 pmol/ml) (Figure 5).

## Discussion

The incidence of squamous cell carcinoma, the second most common form of skin cancer, is rising each year and expected to double in the next 20 years [36]. Reports over the last several years suggest inflammation in the tumor microenvironment contributes to the development or progression of skin cancer [37]–[38]. The persistent recruitment of inflammatory cells found in the tumor microenvironment can enhance proliferation, survival and migration of keratinocytes. Additionally, chemokines, selectins, and their receptors found on tumor cells can facilitate late stages of cancer progression, including invasion, migration and metastasis [37]–[38].

We recently identified a tumor suppressor function of *Tpl2* in skin cancer, with the absence of Tpl2 contributing to both DMBA/TPA-induced tumorigenesis and inflammation [19]. In this model, Tpl2^−/−^ mice showed augmented nuclear factor-kappa B (NF-κB) signaling, neutrophil recruitment, expression of pro-inflammatory cytokines, and edema [19]. The current study was conducted to determine if the enhanced inflammatory response found in Tpl2^−/−^ mice contributes to their heightened tumorigenesis. We found TPA-treated keratinocytes, TPA-treated skin, and papillomas from Tpl2^−/−^ mice have increased expression of COX-2 and its downstream signaling pathways. COX-2, an inducible enzyme involved in prostaglandin biosynthesis, is overexpressed in numerous epithelial cancers [39]. COX-2 overexpression can inhibit apoptosis, while stimulating angiogenesis and invasion, in several cancer types [40]. Additionally, the peroxidase function of COX-2 can aid in the conversion of pro-carcinogens to carcinogens and initiate tumorigenesis [40]. In the case of skin cancer several laboratories have used genetic means to document a cause-effect relationship between COX-2 expression and tumorigenesis [27], [39], [41]–[43]. In this regard, chemically-induced papillomas and carcinomas constitutively overexpress COX-2. Interestingly COX-2 transgenic mice develop skin tumors without the need for a promoting agent such as TPA [39]. In contrast, deleting the *COX-2* gene or treating mice with COX-2 inhibitors suppresses the development or progression of chemically or UV-induced skin tumors in mice [42]–[43]. Consequently, the use of non-steroidal anti-inflammatory drugs (NSAIDs), which primarily work through the inhibition of cyclooxygenases, has been associated with a reduced risk in numerous cancers [44].

We grafted nude mice with v-ras^Ha^ –transduced keratinocytes from Tpl2^−/−^ or wildtype mice mixed with fibroblasts from the same genotype and fed subsets of these animals the COX-2 selective inhibitor Celecoxib to show an upregulation in COX-2 is correlated with increased tumorigenesis seen in Tpl2^−/−^ mice. We found nude mice grafted with keratinocytes and fibroblasts from Tpl2^−/−^ mice developed significantly larger tumors than mice grafted with keratinocytes and fibroblasts from wildtype animals. Administration of Celecoxib to nude mice grafted with keratinocytes and fibroblasts from Tpl2^−/−^ mice decreased the size of the tumors to a level indistinguishable from untreated wild type control mice. Thus, these data implicate COX-2 as a causative agent in the high skin tumorigenesis found in these animals.

Activation of COX-2 results in the conversion of arachidonic acid to PGE_2_. In our report we found production of PGE_2_ was significantly higher (both basally and upon TPA-treatment) in Tpl2^−/−^ keratinocytes than in wildtype keratinocytes. *In vitro* and *in vivo* studies correlate PGE_2_ levels with enhanced keratinocyte proliferation [31], [45]–[46]. Reports also show tumor promoting agents such as TPA directly increase the release of PGE_2_ in keratinocytes, and released PGE_2_ is associated with increased DNA synthesis [45]–[47]. Additionally, treatment with the NSAID indomethacin inhibits TPA-stimulated keratinocyte proliferation but this effect is reversed by addition of exogenous PGE_2_ [45]. Despite having important effects on the normal growth of epidermal tissue, increased PGE_2_ has been associated with tumorigenesis. This is presumably due to the ability of PGE_2_ to activate multiple signaling pathways including EGFR, cAMP, PI3/AKT, and MAPK [31]. Activation of these signaling pathways results in elevated promoter binding to genes such as cyclin D1 and VEGF. This can cause increases in cancer cell growth and angiogenesis [31].

As mentioned earlier, PGE_2_ binds to one of four transmembrane receptors, EP1, EP2, EP3, or EP4. In our report, using both *in vitro* and *in vivo* techniques, we found significantly higher expression of EP2 and EP4 receptors in Tpl2^−/−^ mice compared with wildtype mice. In the case of EP2, TPA-treated keratinocytes, TPA-treated skin, and papillomas from Tpl2^−/−^ mice all had elevated EP2 protein levels compared with wildtype mice. As previously reported, EP2 mRNA is elevated in human squamous cell carcinomas compared to normal skin, as well as in UV-treated skin [29], [48], [49]. Additionally, whereas EP2 -transgenic mice have enhanced skin tumor development, survival, proliferation, angiogenesis, and inflammation, ablation of EP2 leads to a decrease in TPA-induced inflammation and skin tumorigenesis [29], [35]. Moreover, EP2 knockout mice have significant decreases in skin tumorigenesis, keratinocyte proliferation, epidermal thickness, and inflammatory parameters [50]–[52].

In our study, we also found differences in the expression pattern of the EP4 receptor between genotypes. In TPA-treated skin and TPA-treated keratinocytes, wildtype mice have moderate basal expression levels of the EP4 receptor with expression declining after TPA treatment. This is in contrast to the Tpl2^−/−^ mice where expression of this receptor is low at basal levels but increases with TPA treatment, peaking at 24 hours. Others have found increased expression of EP4 in papillomas and squamous cell carcinomas compared with normal and UV-irradiated skin [49]. EP2 and EP4 both function through the same cAMP-mediated pathway. In the current study we found TPA-treated Tpl2^−/−^ cells produce 46% higher cAMP levels than TPA-treated wildtype keratinocytes. EP3 works in an opposing manner to EP2 and EP4 as it can inhibit production of cAMP. Interestingly in our study, EP3 expression increases with TPA treatment in wildtype mice and decreases with TPA treatment in knockout animals. Thus, the induction of EP2 and EP4 in TPA-treated keratinocytes in Tpl2^−/−^ mice, as well as the reduction in EP3 in TPA-treated keratinocytes in knockout animals both may contribute to the enhanced cAMP levels. Overall, increases in cAMP and cAMP-mediated pathways together with aberrant activation of cAMP-controlled genes are linked to carcinoma growth, angiogenesis and resistance to apoptosis [33].

**Figure 6:**
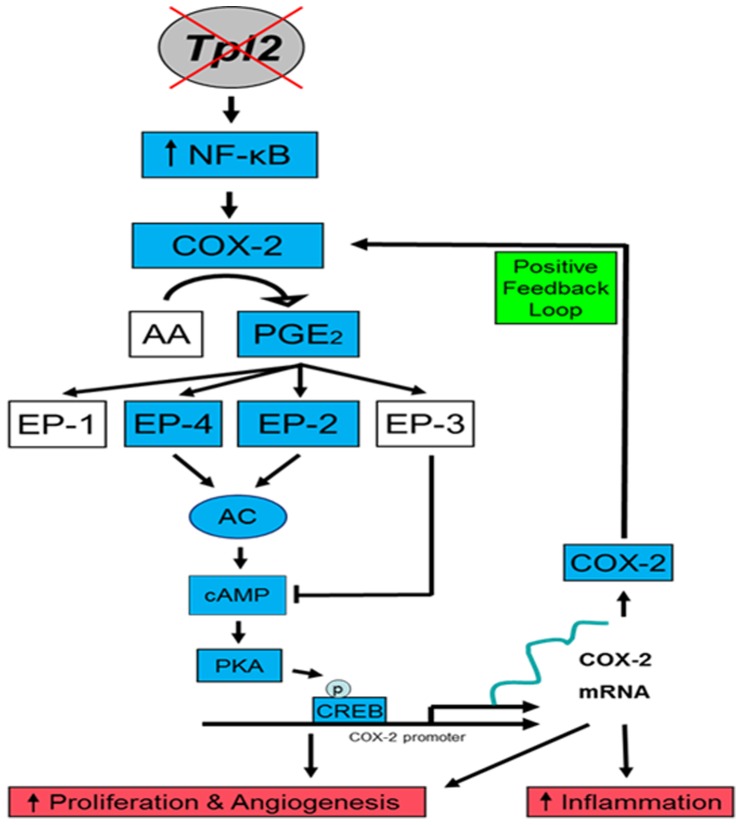
Proposed model describing how loss of Tpl2 results in increased inflammation and tumorigenesis. Tpl2 is normally held in complex with p105 NF-κB, preventing proteolysis and activation of the NF-κB and MAPK signaling pathways. Without Tpl2, NF-κB activity increases (see reference 19), resulting in increased production of NF-κB target genes such as COX-2. COX-2 activation results in the conversion of arachidonic acid to PGE_2_, which then can bind to one of four G-protein linked receptors (EP1–EP4). EP2 and EP4 receptor activation causes the formation of cAMP from ATP. cAMP can then bind to cAMP response genes such as CREB and PKA. The end result is an increase in inflammatory and cell survival proteins, ultimately contributing to both tumorigenesis and inflammation.

In summary, using both *in vitro* and *in vivo* experiments we demonstrated that *Tpl2* knockout mice have elevated cyclooxygenase-2 (COX-2) and COX-2 downstream factors including PGE_2,_ EP2, EP4, and cAMP. These results suggest one possible mechanism to explain how deletion in Tpl2 can lead to enhanced skin tumorigenesis (Figure 6). The notion that inhibition of COX-2 and its downstream factors is chemopreventative has led to the design of COX-2 selective inhibitors. However, there are safety concerns regarding COX-2 inhibitors, as long-term use may predispose to cardiovascular events [53]. As we begin to unveil the factors in the COX-prostaglandin pathway contributing to tumorigenesis it is critical to understand how these factors are regulated. Identifying how Tpl2 and COX-2 interact may lead to new ways to downregulate COX-2, an enzyme often overexpressed in skin cancer, through the modulation of Tpl2.
